# An Enhanced Architecture to Resolve Public-Key Cryptographic Issues in the Internet of Things (IoT), Employing Quantum Computing Supremacy †

**DOI:** 10.3390/s22218151

**Published:** 2022-10-25

**Authors:** Shuhab Shamshad, Farina Riaz, Rabia Riaz, Sanam Shahla Rizvi, Shahab Abdulla

**Affiliations:** 1 Department of Computer Science and I.T., University of Azad Jammu and Kashmir, Muzaffarabad 13100, Pakistan; 2 UniSQ College, University of Southern Queensland, Toowoomba 4350, Australia; 3 Commonwealth Scientific Industrial and Research Organization (CSIRO), Sydney 2122, Australia; 4 Raptor Interactive (Pty) Ltd., Eco Boulevard, Witch Hazel Ave, Centurion 0157, South Africa

**Keywords:** cryptography, quantum computing supremacy, quantum communication, public-key cryptography, Internet of Things (IoT), quantum computing, cybersecurity

## Abstract

The Internet of Things (IoT) strongly influences the world economy; this emphasizes the importance of securing all four aspects of the IoT model: sensors, networks, cloud, and applications. Considering the significant value of public-key cryptography threats on IoT system confidentiality, it is vital to secure it. One of the potential candidates to assist in securing public key cryptography in IoT is quantum computing. Although the notion of IoT and quantum computing convergence is not new, it has been referenced in various works of literature and covered by many scholars. Quantum computing eliminates most of the challenges in IoT. This research provides a comprehensive introduction to the Internet of Things and quantum computing before moving on to public-key cryptography difficulties that may be encountered across the convergence of quantum computing and IoT. An enhanced architecture is then proposed for resolving these public-key cryptography challenges using SimuloQron to implement the BB84 protocol for quantum key distribution (QKD) and one-time pad (OTP). The proposed model prevents eavesdroppers from performing destructive operations in the communication channel and cyber side by preserving its state and protecting the public key using quantum cryptography and the BB84 protocol. A modified version is introduced for this IoT situation. A traditional cryptographic mechanism called “one-time pad” (OTP) is employed in hybrid management.

## 1. Introduction

The Internet of Things (IoT) is becoming increasingly popular in biomedical, academic, manufacturing, and other fields that need an extensive network of microcontrollers. Quantum features such as entanglement and superposition are employed to solve complicated problems. However, there are specific points of contention regarding molding and measuring quantum speed. One apparent challenge is the difference in computing capability between conventional and quantum computers.

The encryption procedure is used to safeguard data-in-transit (communications), data-at-rest (stored), data-in-use (in memory), data integrity (digital signature), and all authentication processes (identity validation). With the advent of quantum computing, it will be possible to shorten the time required to break some of the encryption algorithms currently in use, particularly the asymmetric algorithms (i.e., public key algorithms) that are used to establish communication protocols such as SSL and TLS (used for HTTPS) or to sign information digitally.

Data must be processed in a single binary state in classical computing based on the Boolean logic field of science. Several fundamental particles, such as electrons or photons, can be used to represent zero or one in a quantum computer, depending on their charge or polarization. All these particles’ properties and performance are referred to as a quantum bit, or qubit, in the quantum computing idea [1].

The two most important aspects of quantum physics are quantum superposition and entanglement. Quantum entanglement enables qubits divided over unbelievable ranges to function immediately (not restricted to the speed of light). Although the gap between the associated particles is large, they remain entangled if separated. Significant processing power gain can be achieved by combining quantum superposition and interposition. In an ordinary computer, only one of four binary configurations (00, 01, 10, or 11) is saved at any time; however, a 2-qubit registry will instantaneously store all four qubits, each representing two numbers. When many qubits are used, the capacity increases exponentially [1].

### Quantum Computation

Just as classical computation involves bits, quantum computation uses quantum bits and qubits, usually denoted using “bra–ket” notation as |ψ⟩. The “state-vector” of the qubit is represented by a ket, which is just a vector representation. The equivalent |0⟩ or |1⟩ states of qubits may be like conventional bits with 0 or 1. It is like a linear combination of the amplitudes of probability for each of the kets α and β, where α|0⟩ + β|1⟩ = 1 and |α|^2^ + |β|^2^ = 1 [2].

A quantum machine can read or “measure” a qubit like a computer can read the value of a conventional bit. In the measurement, the qubit’s state is collapsed to one of two values, |0⟩ or |1⟩, depending on the state of the measure. The likelihood of a vector collapsing into one of two states is proportional to the square of its amplitude. Even if the superposition collapse’s precise mechanism is unclear, it is an essential characteristic of quantum mechanics since it was obtained from practical evidence. In the same way that 0 and 1 are binary, these states will be employed for calculation.

Consider the qubit |ψ⟩ = 1/√3|0⟩+ (2/3)^1/2^|1⟩, which has the value 1/3|0⟩ + √3|1⟩. The likelihood that |ψ⟩ will be equal to |0⟩ when measured is (1/3)^2^, which is one-third. This vector formula may be used to define any qubit or state vector that exists. It is stated that the qubit is in a superposition of the values |0⟩ and |1⟩ if |ψ⟩ is a linear combination of |0⟩ and |1⟩ and neither amplitude is zero in this case. In quantum computing, superposition is a fundamental property that cannot be ignored [2]. To modify probability, we need quantum operators known as gates. For example, the Z gate inverts the qubit in the way given in Equations (1) and (2). Similarly, the Hadamard gate, or H gate, performs a “quarter turn”, shown in Equations (3) and (4).

|ψ⟩ = |0⟩ → Z → |ψ⟩ = |1⟩(1)

|ψ⟩ = |1⟩ → Z → |ψ⟩ = |0⟩(2)

|ψ⟩ = |0i → H → |ψ⟩ = 1/√2|0⟩ + 1/√2|1⟩(3)

|ψ⟩= |1i → H → |ψ⟩= 1/√2|0⟩ − 1/√2|1⟩(4)

To test this hypothesis, we may measure |ψ⟩ in this condition where the H gate is applied and α and β both equal 1/√2, and (1/√2)^2^ = 1/2, where it will have the same chance of falling to either |0⟩ or |1⟩ as soon as the H gate is applied.

A basis is a collection of vectors against which to measure, with many different bases. The H gate inserts |0⟩ →|+⟩ and |1⟩ → |−⟩, but it also converts the Hadamard basis to the standard basis: |+⟩ → |0⟩ and |−⟩ → |1⟩. The probability measures of the qubit are given in Table 1.

The standard and Hadamard bases are referred to as orthonormal bases because of their perpendicular relationship. That is, if a |ψ⟩ = |+⟩ or |ψ⟩ = |−⟩ value is measured on the standard basis, it has a 50% probability of being |0⟩ or |1⟩ and vice versa [2].

Quantum computers can solve the DLP on an n-bit integer in O (*n*2log *n* log log *n*) time [3]. As a result, the rising popularity of quantum computers presents a severe danger to the Diffie–Hellman KEP and asymmetric encryption security. The BB84 protocol is a quantum key distribution (QKD) protocol that enables two parties to utilize a verifiably secure channel to co-create a shared key that can then be used to encrypt communications symmetrically.

Scholars debate on many forms of quantum speed and quantum computers, each of which is meant to handle a particular set of problems. Existing cryptographic approaches might be revolutionized by quantum computers, which are expected to appear soon. According to experts and academics who have analyzed technical literature, quantum computers can execute algorithms that enable the decryption of encrypted communications without needing a decryption key. These quantum algorithms, they claim, will make “existing cryptography approaches easier to break”. When these algorithms are broken, the victims are exposed to significant strategic and security concerns.

Although there has been a significant interest in quantum cryptosystems, more studies on their IoT application are still required. This article explains how to create quantum-resistant solutions for the future generation of Internet of Things developers. This system deals with implementing the BB84 protocol using the simulation package SimuloQron. The proposed architecture ensures the security of the Internet and other cryptographic-based systems. It is essential to expand the mathematical analysis to construct a quantum-resistant design for future encryption.

## 2. Literature Review

The Internet of Things (IoT) is a concept in which devices and gadgets connect without requiring human engagement. This happened earlier in the SCADA and ICS industries when conventional networking protocols became accessible through the Internet. For instance, commands can be issued to instruct the use of IP protocols based primarily on MPLS over open communication networks in hundreds of thousands of homes, to guide the connection of intelligent meters or instruct the connection of devices that support smart cities, or to direct the connection of hundreds of thousands of autonomous vehicles on our roads. IoT technology has increased the demand for smart appliances in various industrialized health insurance, logistics support, and agricultural sectors [4]. Data integrity is checked to guarantee that data division performed by globally scattered IoT devices is suitable and effective. Because of such revolutionary infrastructures, new defense weaknesses emerge. The attack vectors’ scale is unparalleled, with a single successful infiltration potentially affecting millions of devices [5].

A single photon is a minimal amount of light that obeys the laws of quantum physics. This means that an eavesdropper cannot measure the value of a photon while allowing the other half to continue its path. In QKD, the two legitimate parties work together to prevent eavesdropping by forcing the eavesdropper to introduce errors. One of the pioneers or founders, Richard Feynman, suggested and demonstrated that quantum mechanical features could be exploited in communication if information bits can be physically described [6]. Encoding transmission of information can be done via electron spin, photon dispersion, or other quantum features.

### 2.1. Quantum-Based Communications

Because of the features of quantum information, quantum communication and information processing outperform conventional communication and information processing in many ways. Quantum information attributes include, but are not limited to, the concept of uncertainty, the non-clone quantum theory, quantum teleportation, and hidden quantum information traits that may be exploited for resistance attacks during cyberspace transmission [7]. The main idea of the principle of uncertainty is the impossibility of determining the particulate position in the micro-world. German physicist Heisenberg introduced the uncertainty principle in 1927 [8].

The unclosed and undeleted characteristics of an unknown quantum state are quantum non-cloning theory. Cloning means that another system can produce an identical quantum state. Researchers have shown that machines cannot replicate quantitative approaches [9]. The undeleting principle may ensure that the enemy’s removal and damage of quantum information are reflected in the secure communication of security and communications networks. In nature, it was suggested that linearity in quantum theory is not permitted to delete a copy of an arbitrary quantum [10].

### 2.2. Quantum Teleportation

The sender measures the quantum state of the original, which the sender classically communicates. Quantum information is the remaining information not extracted in the measurement by the sender and sent on by metric measurement to the recipient. In 1993, an unknown quantum state was proposed to be introduced into televisions [11]. Quantum information has features that classical details do not have. Only standard measurement can expose the quantum code’s information while the quantum code is in its entangled state, and this information cannot be accessed by local measure [11]. While desktop quantum code breakers are no longer available, quantum ciphers may still be bought, putting defenders one step ahead of attackers. Symmetric-key cryptography is theoretically secure if a few conditions are met. The single significant drawback to this strategy is the key exchange between Alice and Bob, which requires frequent contact or a considerable investment in infrastructure (e.g., mobile cellular networks).

The non-cloning theorem results from the postulates ensure this criterion is met by quantum key distribution methods such as BB84 and B92 and their management [12]. Previously, conventional computer information could be copied without limitation. Although this quality is usually good, it can be dangerous in some instances (e.g., quantum information has features that classical details do not have). The orthogonal nature of the classical states |0i and |1i makes them simpler to identify. The non-cloning theorem allows quantum computing to separate only orthogonal and known states [13].

Since no computer hardware can distinguish between two nonorthogonal qubits, this problem exists. Continuous-variable quantum key distribution techniques may be implemented since existing key distribution systems employ coherent states rather than single photons. The authors then move on to another key communication challenge, capacity, while we possess the comforting answer for future secure communications in our hands. Entanglement-assisted classical capability enhances the degree of independence by permitting entangled (i.e., correlated) states at the input encoder and joint measurement at the receiver end. Although this technology can potentially increase power, the size of the gain is uncertain.

### 2.3. Public-Key Cryptography and Quantum Computing

Cryptographers manage the power to hide and unhide information using a “key”. The terms “symmetric key” and “public key” are sometimes used interchangeably. Since the emergence of quantum parallelism, quantum computers have performed far better than conventional computers. No matter how fast a computer can process information, the processing capacity of quantum computers will be restricted due to physical considerations. When constructing an algorithm, we must consider its spatial and temporal complexity. This requires not only large-scale quantum computers but also a reasonably lengthy quantum coherence period. If the above two requirements are not satisfied, the operation cannot be completed.

Shor’s algorithm reduced the processing cost of significant integer factorization to a polynomial level [14]. This complexity is equivalent to the RSA public-key cryptography protocol’s encryption and decryption, demonstrating that the RSA is vulnerable in universal quantum computing. Other quantum algorithms, such as Grover’s search algorithm [15] and its improved versions, as well as the Harrow, Hassidim, and Lloyd (HHL) algorithm, display various types of higher speeds for addressing many difficulties [16]. Because of the enormous processing capability of quantum algorithms, researchers have begun to hunt for suitable physical devices for quantum computing implementation. The public key encryption strategy must be modified regularly due to the rapid growth of quantum computing technologies. As a result, cryptologists are continuously on the lookout for public-key protocols that can survive quantum computing assaults, resulting in post-quantum cryptography creation.

Traditional cryptographic algorithms are unsafe or need larger key sizes if large-scale quantum computers are on the market. The impact of quantum computing technology on the conventional cryptographic algorithm is explained in Table 2, given below. Public-key cryptosystems and symmetric cryptography cryptographic systems are commonly utilized. Since the first successful encryption methods were made public in the 1970s, public-key cryptography has been critical for modern Internet communications due to its capacity to give high security [17,18].

This has resulted in the wide spread of public-key cryptosystems such as RSA, ECC, and DH [19]. They are now included in critical Internet protocols such as the TLS, which conventional computer systems and connected devices use to communicate with one another. On the other hand, recent technology innovations and telecommunications have simplified the computing work required to crack asymmetric systems, raising the suggested minimum key size. For instance, since 768-bit and 1024-bit RSA implementations were compromised in 2010, the minimum recommended key size for RSA is between 2048 and 4096 bits (depending on the protected information type). Expansion of the key size is a stopgap measure until technology catches up and delivers the required computing effort [20,21].

If the present state of technology allows for the development of great large-scale quantum computing devices, we may explore the performance of the Shor algorithm on these systems. The time complexity of the conventional cracking strategy on RSA is roughly Ex (O (log N) e^(1/3)^ (log N) e^(2/3)^), while the time complexity of the modern cracking approach on RSA is approximately O (log3 N). Because Shor’s factoring algorithm has an O (log3 n) temporal complexity and is implemented in a neighbor-only, two-qubit-gate, competitor-like (NTC) architecture, the quantum algorithm of Shor poses a significant threat to the security of public-key-encrypted RSA encryption keys.

A classical computer may have a clock frequency of around 10 GHz, implying that the gate speed is approximately 0.1 ns. The trapped ion system and superconducting circuits are, without a doubt, terrible. This can only be done in a 10 s and 20 ns quantum operation. After considering quantum-resistant solutions, the National Security Agency (NSA) suggested in 2015 that the Suite B group’s ECC security be increased. Current public-key cryptosystems are vulnerable to quantum computing, according to the NSA. Quantum computers, according to a new *Technology Review* study, will be able to readily break sophisticated cryptosystems within the next 20 years [22]. The impact of post- and pre-quantum security levels for symmetric- and public-key cryptography is explained in Table 3, given below. Some slandered cryptographic algorithms need larger key sizes, while many of them can be broken easily by quantum computing technology.

In previous studies, the researchers used different techniques to improve public-key cryptography. Keshavarzian suggested an improved deep residual network model for human activity identification based on IoT technologies. Using a range of smartphone sensors, the human body signals were recognized and analyzed on the cloud computing platform. Moreover, the authors proposed a function-as-a-model for real-time measuring activity in the cloud. The suggested approach outperformed various state-of-the-art decision-making methods [23]. A secure IoT-based network architecture based on blockchain technology was proposed in [23] for hybrid industrial applications. According to the authors, the benefits of IoT-based service delivery include cost-effectiveness and precision. Blockchain technology was used to ensure real-time data and guarantee transparency among industrial users.

In 2019, Thigale et al. introduced a breakthrough in IoT, namely a framework for safeguarding data transfer; the authors offered an IoT protocol resistant to cross-layer assaults. Moreover, the system was designed to deal with time constraints and accessible delivery [24]. In 2019, J. Cao created a quantum-resistant access authentication and data allocation approach for large-scale Internet of Things networks. The suggested approach decreased network bandwidth while offering the highest security and privacy against quantum threats. The proposed model was evaluated in real time and yielded the best results [25].

The intelligence service and its analysis can be improved by combining quantum cryptography, ML, and AI techniques. These intelligence services claim to be able to decrypt 2048-bit RSA encryption in 8 h or less, a job that would take the fastest supercomputers in the world roughly 300 trillion years to perform using brute force. Quantum computers may need over 20 million qubits. This field’s developments indicate that such machines might exist in 25 years. If results in quantum decryption outpace progress in quantum encryption, there is a possibility that malicious use of such computers might endanger national and international security because of the reduction in duration from millions of years to a few seconds [26,27].

The National Quantum Initiative Act of 2018 established a coordinated government initiative with USD 1.275 billion in financing over five years to speed up quantum research and development. Additionally, it defined the duties of the National Quantum Coordination Office, the National Quantum Initiative Advisory Committee, and the National Science and Technology Council Subcommittee on Quantum Information Science. Notably, funding in 2019 and 2020 exceeded the budget set by Congress, demonstrating the importance placed on quantum research and development by the United States [28].

Rosa M. Gil Iranzo discusses the drawbacks of interfaces for quantum computing that make it easier to master the new paradigm. The author suggested a layer to establish appropriate learning conditions for carrying out computations without enhancing mastery of the fundamental ideas of quantum computing. The emphasis of planned work is human-centered computing, which will support levels such as high school, university, and research. This study uniquely integrates science and technology to build interfaces for quantum computing [29].

U. Chukwu used two quantum-ready techniques, quadratic unconstrained binary optimization (QUBO) and constrained-optimization sampler, to tackle the NP-Hard graph issue of graph partitioning. Both methods frequently produced better partitions than the standard graph partitioners designed for that specific purpose [30].

The idea of quantum computing has advanced to the point that it is no longer considered science fiction. As they are entirely new fields, quantum clinical medicine and quantum surgery have yet to reach their total growth and potential. These fields are conceptual extensions of quantum computation and many body systems. To allow these fields to ultimately materialize and mature into secure clinical applications that benefit humanity, novel formalisms and methods must develop [31].

As a result, the cybersecurity sector is preparing for future development by using cutting-edge technologies such as AI, quantum computing, blockchain, and data science. Quantum computing is an emerging field that uses the ideas of quantum mechanics and combines them with computer science, physics, and mathematics to accomplish calculations. This new computing technique can solve various complex scientific problems and open new possibilities. Soon, cybersecurity infrastructure will be rendered obsolete by the development of futuristic technology [32].

## 3. Methodology

Quantum computing provides a new and powerful toolset that has the potential to collapse many cryptosystems. Anything transferred across an observable network is vulnerable to an adversary without quantum-safe encryption. Quantum computing can encrypt data encrypted in the past or conveyed in the future. It may be conceivable to develop a quantum computer soon; nevertheless, the time it takes to upgrade the current IT infrastructure is more important. It is critical for companies interested in keeping secret information safe from adversaries to take a proactive approach to information security. This requires considering the time needed to maintain and update protection over a long period. Not all security systems and cryptographic technologies are vulnerable to quantum attacks. Today’s believed quantum-safe surveillance may be susceptible in the morning.

Quantum computers are very prone to security safeguards that can be broken in seconds. As a result of AES’s ability to overcome a quantum computing flaw by increasing the size of its key, it is called quantum-safe. Because they cannot increase key sizes at a rate fast enough to keep pace with the exponential rise of quantum computing, RSA and ECC ciphers are not quantum-safe. It takes a regular computer two years to process an 8-bit RSA or ECC key. On the other hand, a 16-bit RSA or ECC key can be processed every two years. Symmetric-key encryption methods such as RSA and AES are commonly considered impervious to quantum attacks. When public-key cryptography is preferred over symmetric-key cryptography, the employment of quantum-safe cryptographic ciphers is necessary. Examples of public-key algorithms include RSA, ECC, Diffie–Hellman, and DSA.

### 3.1. Quantum Cryptography

Quantum cryptography is an intriguing area that utilizes quantum physics to create the world’s most secure cryptosystem. Quantum cryptography is based on the utilization of photons and their basic quantum features to create an unbreakable cryptosystem since it is impossible to determine the quantum state of any system without alerting it. Nobody can breach it without the sender or recipient of the communication being aware. Quantum cryptography is based on the use of nature’s tiniest individual particles, photons. These photons possess the ability to exist in several states concurrently, and their circumstances change only when they are measured. This is the primary characteristic that quantum cryptography techniques exploit. When a message travels across a channel from sender to receiver and a hostile party attempts to capture the transmission, the sender or receiver instantly notices the change in the status of the photon. Additionally, there is a form of strategy that takes advantage of a quantum entanglement characteristic. Quantum entanglement is a phenomenon in which, even when a physical distance separates two quantum particles/photons, every change in one photon results in a change in the other, making it easier to identify an intruder in a network.

### 3.2. Quantum Key Distribution (QKD)

Since quantum computing conveys data through a stream of photons, quantum key distribution is fundamental in quantum cryptography. These photons have what is referred to as a “spin”. Spins are available in the horizontal, vertical, 45° diagonal, and −45° diagonal directions. Rectilinear filters are defined as horizontal and vertical, whereas diagonal filters are defined as horizontal and vertical. Horizontal filters represent binary 1, whereas vertical filters represent binary 0, as do 45° and horizontal ones −45°. The Heisenberg uncertainty principle, a fascinating concept in physics, argues that we cannot measure all the characteristics of a particle without altering its present state. This same idea holds for photons. If we attempt to measure the spin of photons, the spin will vary, affecting the photon’s value. Thus, we may deduce that an unwelcome object has disrupted the stream of communication photons [25]. A stream of polarized photons is sent to Bob by Alice, who randomly chooses one of the polarizations. Bob chooses at random between + and x bases after receiving a photon. After the photons have been measured, Bob will transmit the command sequence he used to Alice. These exchanges will be entirely open to the public. When Bob inquires, Alice reveals which grounds she used are comparable [33].

Bob may, on average, estimate the correct basis with a probability of 50% and therefore obtain a polarization comparable to that supplied by Alice. The critical step is next to interpret the remaining photons in the sequence as 0’s and 1’s. Eve will overhear the communications between Alice and Bob about the base sequences they employed and will ascertain if Bob correctly predicted. However, this provides no information about the key since Alice’s polarizations were picked randomly. Assume Bob considered + to be the proper polarization. In that instance, Eve cannot know whether Alice delivered a 0 or a single polarized photon and does not know the key bit represented by the photon. Once Eve has determined the state of a photon, its state is adjusted to conform to the basis Eve employed. Thus, Bob may obtain the wrong end on a similar premise and incur a 50% mistake; Eve’s measurement introduces a 25% inaccuracy [34,35].

### 3.3. Cryptography That Is Not Dependent on the Device

An attempt is made to keep the original quantum key distribution safe even when utilized with insecure third-party devices via device-independent quantum key distribution. Quantum key distribution exchanges a conventional cryptographic key between two computers, Alice and Bob. It is well established that the BB84 protocol (the quantum cryptography protocol) is secure even in the presence of channel noise and probable detector defects at Bob’s end if the device used to generate photons at Alice’s end works flawlessly. However, as we work, this assumption falls apart since there is a significant probability of defective equipment on Alice’s side, jeopardizing the security of the private string shared by Alice and Bob for communication [36].

We will need some devices that can self-test to get to the bottom of this issue. Having passed these tests, the machine is deemed secure for communication use. It is also possible to cross-check polarization and probability distributions. There are a variety of ways to solve these issues. Quantum computers have been shown to solve the fundamental mathematical problems that ensure the security of current encryption, such as DLP and prime factorization. Although symmetric encryption is far less vulnerable to this attack, it is not widely employed due to key distribution issues. Quantum mechanics is one possible answer. Quantum key distribution (QKD) applies to a key distribution technique based on quantum mechanics, and quantum computers are indubitably secure. It enables two parties to produce a symmetric key safely [37].

This study examined the BB84 quantum key exchange mechanism for exchanging encryption keys in this research. Alice and Bob are two parties. The encryption key is shared through a quantum computer qubit-based channel. An eavesdropper cannot intercept this transmission to acquire any information without disrupting or measuring the qubits introduced. Since the foundation for encoding information is uncertain, noise is introduced into the signal. We demonstrate that Alice and Bob can discover an eavesdropper due to a spike in the error rate of sent data.

### 3.4. Quantum Cryptography in IoT—An Implementation

Numerous security issues exist in IoT devices, threatening the devices, users, and network security. The current classical architecture of the Internet of Things offers no provision for identifying an eavesdropper in the communications channel [38]. The flaw may not be discovered until later, at which point a significant quantity of data may have been communicated to any malevolent actor. Certain viruses may infect systems, so restarting the systems is the only way to eradicate the viruses. For an extended period, the industrial and business plans will be on hold. As a result, there are many points of weakness, and IoT systems are very vulnerable to attack [39].

A significant feature of quantum cryptography is the distribution of quantum keys, as described before. The most outstanding characteristic of the quantum key distribution is the channel’s capacity to identify the existence of an eavesdropper inside the system’s design. This is in stark contrast to traditional cryptographic algorithms. There are various variants of the quantum cryptography protocol BB84 [40]. However, the primary difficulty in implementing these protocols physically is the most significant distance that photons may travel. Photons are light particles, yet environmental or natural tragedies readily distort them.

These distortions are not something that any corporation can afford. The current quantum key distribution mechanism is optimized for use with two devices. This is not achievable with present IoT systems, which rely on the communication of hundreds of devices. Thus, we may propose a method combining conventional and quantum techniques to resolve these issues. One idea is to employ quantum technologies to produce a long and unique cryptographic key for each device while retaining the present semiconductor chips. Quantum random number generation (QRNG) is a technology that generates highly unpredictable noise and may be used to achieve this goal. Quantum computing is capable of effectively and quickly creating such huge quantities. To make things more complicated, each gadget will have a unique code. Obtaining the key requires access to the device’s settings, which is very difficult to do without being seen. Thus, the key may be safeguarded, and confidential communications can be maintained. Moreover, device-independent quantum cryptography may be used to verify the trustworthiness of built devices. It is possible to employ device-independent quantum cryptography to verify that the manufactured devices are trustworthy.

### 3.5. BB84 Protocol

**Figure 1:**
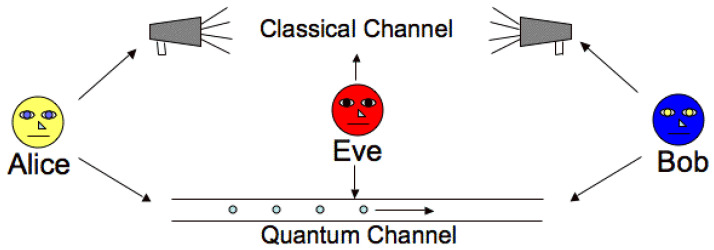
BB84 protocol.

The BB84 [41] protocol is one of the first and most well known quantum data encryption methods. The protocol is usually participated in by two participants, known as Alice and Bob. The third party trying to rob these personal data from the communications platform is called Eve. Each bit of the secret key should be coded into a single photon’s polarizing state (see Figure 1). Because this information will be fragile and unavailable to the eavesdropper, she must discover it. However, when she detects the photon, she must either expose herself or send it again—but she always sends a photon with a false polarization state. This leads to mistakes, and the eavesdropper is revealed. Alice creates two random bit sequences, a and b, each with a length of at least N = 4 L bits, where l is the desired key length to initiate the quantum key exchange. While N does not have to be 4 L, it enables l bits to check the key’s integrity since about half of the bits are wasted during the key exchange. Alice then encodes a into an N-qubit block, |Ψ⟩. This is accomplished using two bases: in our example, the standard and Hadamard bases. The basis on which each bit is encoded is decided by the corresponding bit in b, with bi = f0 or 1 g, where 0 denotes the standard basis and 1 denotes the Hadamard basis. As a result, each qubit is either on the standard or Hadamard basis and is in one of the four states shown in Table 4.

Next, Alice sends each qubit to Bob using a public quantum channel. After Bob has each qubit, he may build the whole qubit block |Ψ. Assuming a flawless quantum channel and the absence of eavesdropping, there should be no disruption or noise in the transmission; hence, |Ψ⟩ = |Ψ⟩. After Bob receives all qubits, he converts each qubit in j i_0_ to a bit sequence a_0_ using a random measurement basis for each bit. The selected bases are stored in a bit sequence denoted by |Ψ⟩. Bob then notifies Alice that he has quantized all the qubits she has received. Because Bob has a 50% chance of selecting an inaccurate measurement basis for each qubit and a 50% chance of measuring the correct value using the faulty measurement basis, Bob has estimated 75% of the qubits correctly on average (see Table 5).

While 75% of bits are measured accurately on average, 50% of qubits are measured correctly on a guaranteed basis. Alice has (a, b), and Bob has (a_0_ b_0_), but none are aware of the others and a_0_. Alice and Bob now swap their respective bases, b and b_0_. Alice and Bob delete any bits encoded and measured in distinct bases: bit I from a and a_0_ is discarded if bi! = b_0_ i. They store the leftover bits from a and a_0_ in two new bit sequences, k and k_0_. Both Alice and Bob now possess the same key, k = k_0_. They exchange a random number of bits from k to ensure that their key creation is error-free. If the bits transmitted are identical, Alice and Bob may be confident that there was no eavesdropper and that their key is safe. They may now use the key to communicate symmetrically encrypted or even an OTP on a traditional channel.

If an eavesdropper, Eve, were to listen in on the discussion, she would be unable to glean any meaningful information from the qubits since she would not be aware of the basis on which the qubits are encoded, and she would also be unable to reproduce the qubits. As a result, her sole option is to conduct a “man-in-the-middle” assault in which Eve impersonates Bob to Alice, and Alice impersonates Bob. Eve listens in on the quantum channel and waits for Alice to send qubits to eavesdrop on the conversation. While Alice transmits the qubits to Bob through the quantum channel, Eve intercepts each qubit and creates her qubit block |Ψ⟩. She then re-encodes them using the same bases into |Ψ I and forwards the qubits to Bob. With the qubits now in Eve’s possession, she attempts to measure the qubits or clone them on either of the two bases randomly. However, as previously shown, this would introduce noise to the signal, reducing Bob’s correct average measurement.

The average proportion of accurate answers is (2 × 100% + 6 × 50%)/8 = 62:5% average. When an eavesdropper is present, at least one-party measures half of the bits included in the produced key erroneously, and the proportion of bits successfully measured decreases from 75% to 62.5%, leaving just 25% of qubits calculated on the same basis as during encoding. Once the bits that Bob measured differently from Alice are deleted, the probability of Bob measuring any qubit remains only (2 × 100% + 250%)/4 = 75%. As Alice encodes the same value, about 25% of the bits in k_0_ are wrong. When Alice and Bob exchange some bits to check their accuracy, even if just four bits are exchanged, they will both discover on average that the qubits were measured maliciously during transmission, at which time Alice and Bob may terminate communication.

In practice, this protocol can be implemented using polarized photons as qubits, which can be sent between Alice and Bob using fiber optics. The data are encoded into the photons using the polarization angle since photons can act as a qubit. In this case, the bases for encoding data are the standard and the Hadamard bases; however, all that is required to perform any QKD protocol is the ability to communicate qubits over a public channel with a shallow error rate. Meanwhile, the principle of uncertainty is based on measurement by qubits. There is a new definition. Various foundations are used to evaluate qubits. It is up to the person doing the evaluation. As per the uncertainty principle, the general basis value (|0⟩ and | one⟩) of a simple qubit will be different from the sign basis value (|+⟩ and |−⟩) or any other different basis. There are several ways in which the BB84 protocol safeguards a user. The crucial factor is that the key will be sent over a quantum channel.

The random number and quantum base format are converted to a qubit by quantum mechanism techniques of qubit generation. The sender (Alice) performs this process and sends the qubit to the receiver (Bob). The receiver (Bob) is responsible for the second step. Bob generates the check bits after guessing the random quantum basis and a binary format of random numbers. He (Bob) sends the sender his check bits (Alice). Alice compares her qubit to Bob’s check bits in the third phase. After the comparison, Alice discovers the matching bits that were used to frame the secret key. Finally, Alice uses an XOR operation to prepare the private key value for the cryptography procedure by combining matched and odd bits of Alice’s qubit. Alice uses the communication channel to communicate a matched bit and not compare bit information with Bob. Based on Alice’s input, Bob now determines the secret key value.

### 3.6. Proposed Network Architecture

**Figure 2:**
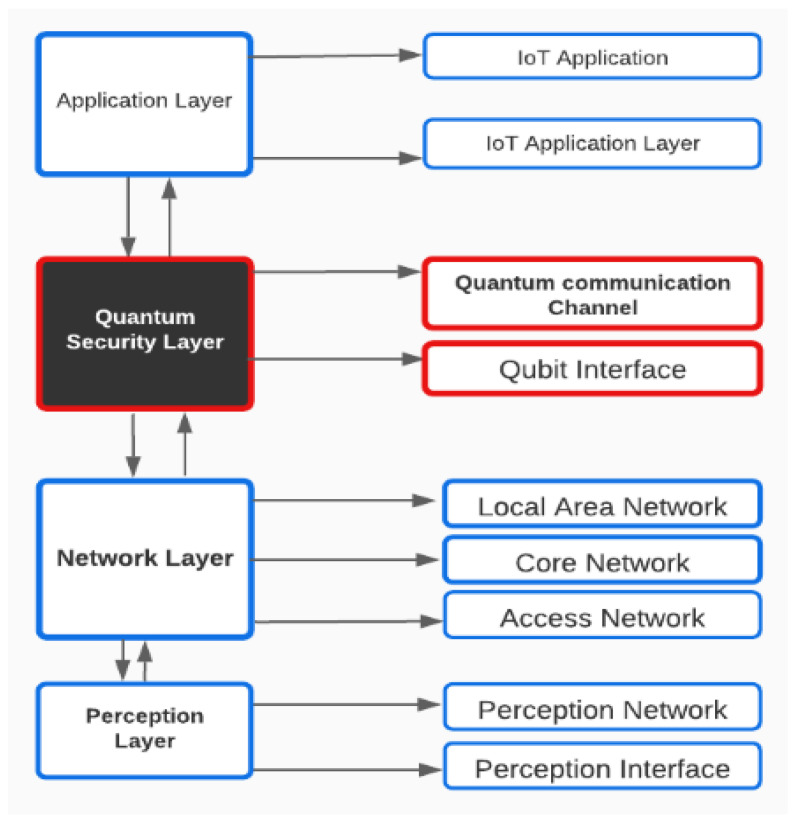
Proposed network architecture abstract diagram.

There are three layers in an IoT system (perception layer, network layer, and application layer). The main goal is to ensure security on all levels. The quantum security layer is a novel addition to the hybrid IoT network architecture system. This layer also sets up and leads quantum cryptography to manage the whole quantum communication channel. The primary purpose of this channel is to safeguard the security key. The additional layers interact with one another in the same manner as before. This layer is the one that appears before the application layer (see Figure 2).

The attacker cannot decipher the plaintext from the ciphertext due to a lack of information. A mathematical problem that is too tough for an adversary to solve is required to derive the plaintext from the ciphertext. The laws of physics prohibit the attacker from learning the data necessary to rederive the plaintext without causing detectable damage to the system. The “informational” paradigm is used by OTP, while AES uses the “computational complexity” paradigm. QKD and symmetric encryption, on the other hand, remove the protection provided by the assumption of computational complexity, making it vulnerable to attack by anybody who can solve the mathematical problem connected with symmetric encryption. Combining QKD with a symmetric cipher provides a solution to the worst of both worlds in terms of implementation problems (not to mention possible side-channel attacks).

This results in a system that is no more secure than traditional cryptography. It is possible to obtain a more secure system than standard cryptography if one does not use a symmetric cipher and instead exclusively uses the distributed bits with the plaintext bits (analogous to what an OTP does). Different questions remain, such as whether it is more effective (because of the side-channel attacks seen in QC implementations) and if the higher implementation costs are worth the additional strength.

## 4. Implementations and Results

This proposed model has been created to enable future expansion. It has two primary classes in two direct files: sender and receiver, responsible for controlling the whole execution. They are responsible for the quantum key distribution procedure and, ultimately, the message exchange. Each BB84 procedure and post-processing stage is separated to ensure responsibility separation. Additionally, a second file is used to implement the eavesdropping attack.

### 4.1. Software Requirements

Linux OS: Linux is an operating system (OS) for personal computers (PCs), servers, mainframe computers, mobile devices, and embedded devices that is free and open-source, developed by the Linux community.

SimuloQron: The end nodes of a quantum internet are a few qubit computers, which may exchange qubits utilizing a quantum internet. Specifically, SimuloQron enables the installation of local simulation software on each computer in the network that gives the appearance of a local quantum processor to possible applications. The local simulation programs on each classical computer communicate with one other classically, establishing a simulated quantum internet permitting the interchange of simulated qubits between the various network nodes and the production of simulated entanglement. SimuloQron must already operate locally or at a recognized address before a client can use the CQC. Since all quantum actions are conducted on the server, a connection object must be created initially.

Python: Python is a high-level programming language that can be interpreted, dynamic, and object-oriented. In addition to promoting readability, Python’s concise and easy-to-learn syntax also helps to reduce application maintenance expenses.

The CQC Interface: To communicate with a SimuloQron server, programs must use the CQC interface library. Command pattern implementations are available in Python, C, and Rust for client and server implementations. The Python library will be examined in depth for the sake of this study. Users may produce and modify qubits using the CQC client-side library, making CQC very user-friendly.

### 4.2. Simulation without Eve

Assuming ideal qubits, we do not consider any noise in this simulation. On the other hand, we also believe that Eve cannot change public communications validated using message authentication codes.

The results of the simulation meet the theoretical expectations (Table 6) for 10-qubit random values by Alice and Bob. Additional examples of the model’s execution are provided in the following section.

**Figure 3:**
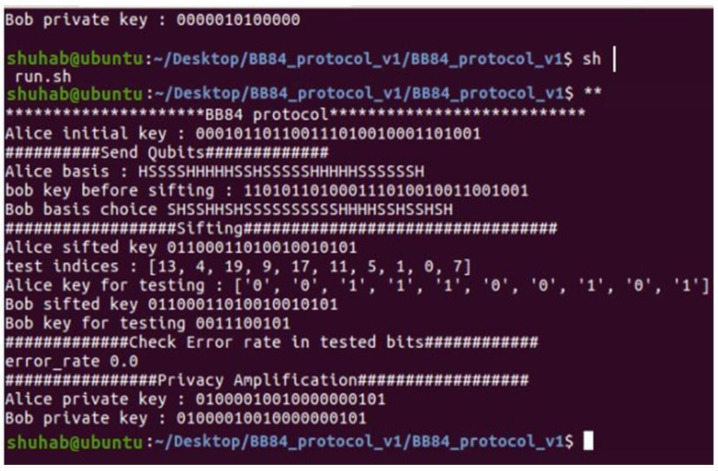
Execution of the proposed protocol.

**Figure 4:**
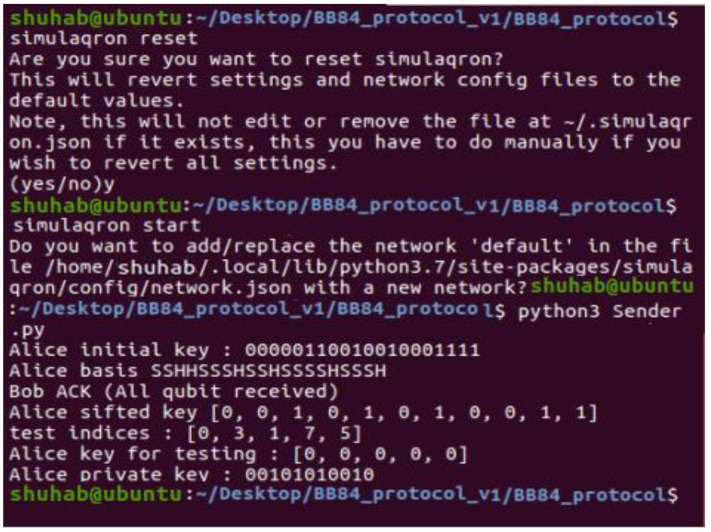
Execution of the Sender.py.

**Figure 5:**
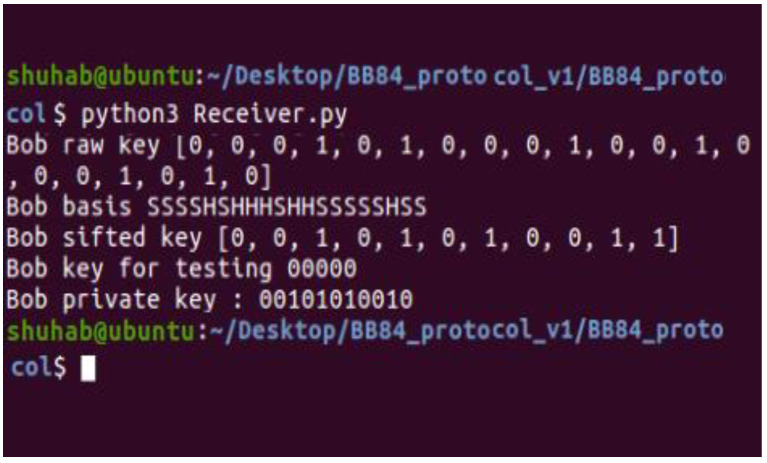
Execution of the Receiver.py.

Figure 3 depicts the execution of the whole protocol in the same Linux terminal as in the previous figure. Figure 4 and Figure 5 illustrate the implementation of two files (Sender.py and Receiver.py) on two terminals, one at a time, in two different terminals.

### 4.3. Explanation

The following is a description of how this simulation worked:

Data encoding: This stage encrypts the encryption key’s bit values using qubits. For each bit with a value of 1, we apply an X-gate. However, no extra gates for zeros are required since each qubit in SimuloQron is initialized to |0⟩.

Choose a random basis: The sender and receiver choose a random basis by applying Hadamard gates to a random selection of qubits. The simulation depicts Alice and Bob’s fundamental decisions. The Hadamard and standard bases are denoted by “H” and “S”, respectively.

Confirmation: Bob sends a message to Alice confirming that he has received all the qubits (Figure 4, Bob Ack). Then he sends the list of his basis choice to Alice (using the classical channel).

Key sifting: Both parties perform key sifting by selecting only rounds in which they have chosen the same basis. This step is also shown in the simulation (Alice sifted the key, and Bob sifted the key).

Testing: Bob now selects a set of test rounds (pairs of measurement basis) and sends them to Alice via the classical channel to compare. Then, Alice computes the error rate in the tested bits. If the error rate is equal to 0.0%, both parties continue the execution of the protocol (Figure 4).

Privacy amplification: Alice and Bob perform privacy amplification to extract the final private key. At this point, Alice generates a random seed and sends it to Bob. Both parties XOR each bit of this seed with each bit of the raw key. After performing the XOR operation, they obtain their private shared key. There are other more secure methods to extract the final secret key, but we only consider the XOR method for this simulation.

### 4.4. Simulation with the Presence of Eve

**Figure 6:**
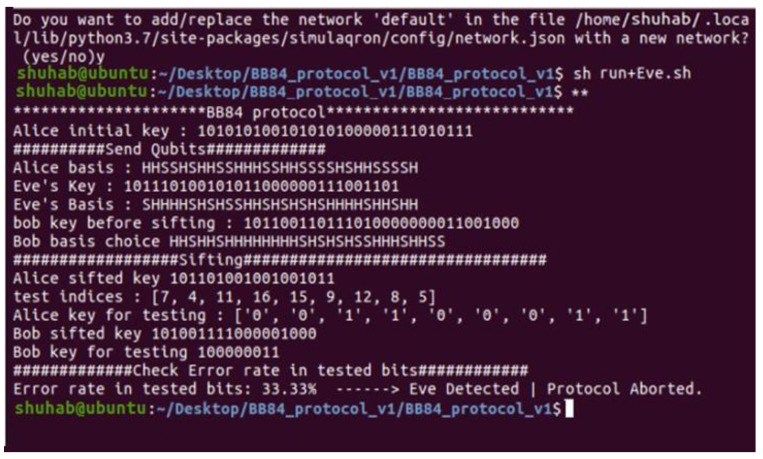
Testing of error rate in presence of Eve.

Eve measures each qubit on the standard or Hadamard basis at random. In the rounds in which Alice’s and Bob’s measurements match, Eve has a 50% chance of using the correct basis, which translates into a 50% error rate in the test bits, and the raw key resends them on the same basis. In this case, the protocol is aborted because the error rate exceeds the threshold (the threshold is equal to 0 because the simulation does not consider noise). This indicates that Eve tampered with the transmissions and projected the qubit into a different state. In conclusion, Eve is detected, and the protocol has been tampered with (Figure 6).

### 4.5. Sending Messages

**Figure 7:**
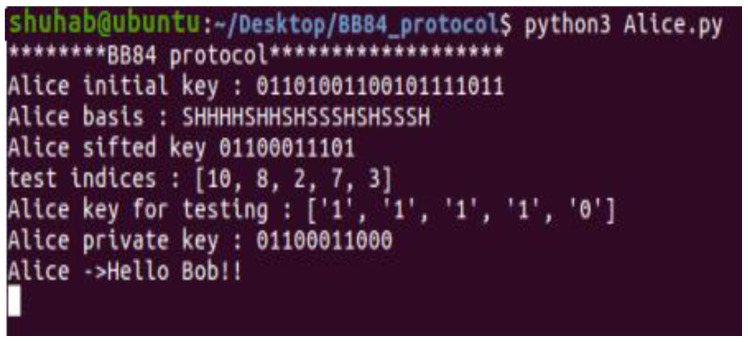
OTP sender (Alice).

**Figure 8:**
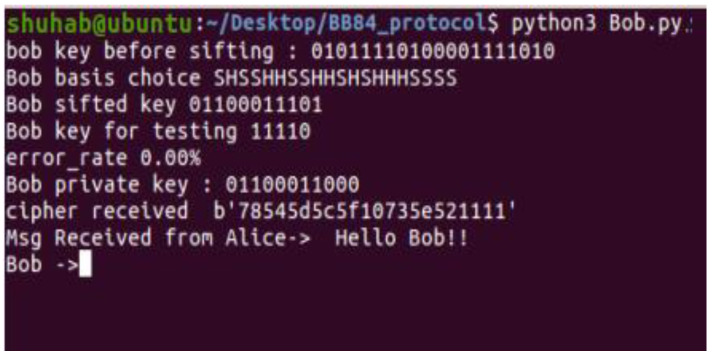
OTP receiver (Alice).

In this example, Alice sends the message “Hello Bob!” to Bob. The sender uses the BB84 final key and classical one-time pad to encrypt the message (Figure 7). Bob receives the encrypted message. He decrypts the message using the same shared key. As shown in Figure 8, the receiver successfully decodes the message and obtains the plain text “Hello Bob!”.

Notice that each key should be used once, with each message encrypted with the one-time pad.

**Figure 9:**
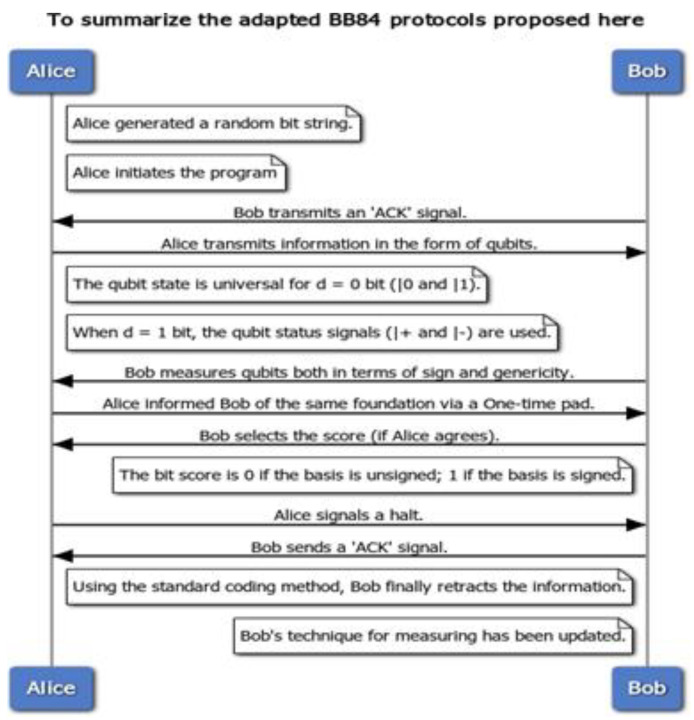
Adopted BB84 protocol.

Assume the package is labeled with the word “apple”. Its score will be “0 15 15 11 4”. These scores are calculated by ranking “A-Z” on a scale of “0–25”. Assume that the sample code is “Abcde”. In this case, “0 1 2 3 4” is the first crucial core. It is referred to as a “one-time pad”. This key will be provided to the receiver over a secure conventional communication channel that will be included in the data encryption step. That secret key will vary when the “BB84 protocol” is followed. Alice’s qubit will be a deciding bell state for one packet of transmitting the information. Because the total probability value of such states is cos2θ + sin2θ, a and b, two balanced variables, may be integrated. As a result of this, (a^2^ + b^2^) = cos^2^θ + sin^2^θ. Because a = 1/2 and b = 1/2 in this equation, the probability value is not a difficult option. It will be either (1/√2)2 or 1/2. The entire system can be based on various entangled states by changing the importance of a and b while keeping the normalized conditions. This key value will now take the form of an angle. There is an angle pattern that Bob may employ to compute the qubit. This angle indicates the relationship between any orthonormal basis “(U, U⊥)” and the generic orthonormal state “(|0(|1))”. Furthermore, after each transmission sequence, this sequence will change. The pseudo-code for the adopted BB84 protocol is given in Figure 9.

**Figure 10:**
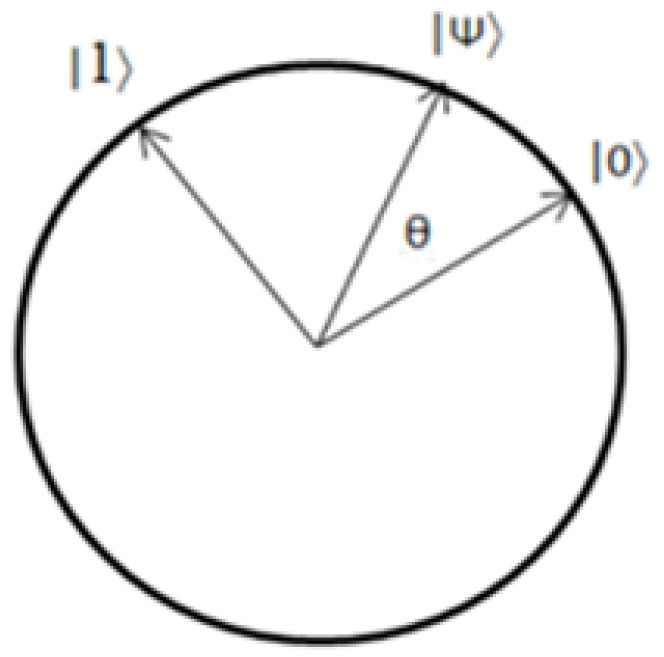
Quantum state |Ψ⟩ after rotation of θ in Hilbert space.

Assume a 0 bit was transmitted. Alice recognizes that the value provided to it is one of the following: “(1/√2) (|++⟩ + |−−⟩), (1/√2) (|++⟩ − |−−), (1/√2) (|+−⟩ + |−+⟩), (1/√2) (|+−⟩ − |−+⟩)”. If the measurement is made at a 45-degree angle, Bob examined the slopes of 0, 45, and 90 degrees. The angle is 45 degrees, as Alice confirmed to Bob, and Bob is aware of the data bit, which is zero. Traditionally, mail confirmation is performed on a one-time pad. This sequence (0, 45, 90) will now be modified, as shown in Figure 10, so that only Alice and Bob know it.

**Figure 11:**
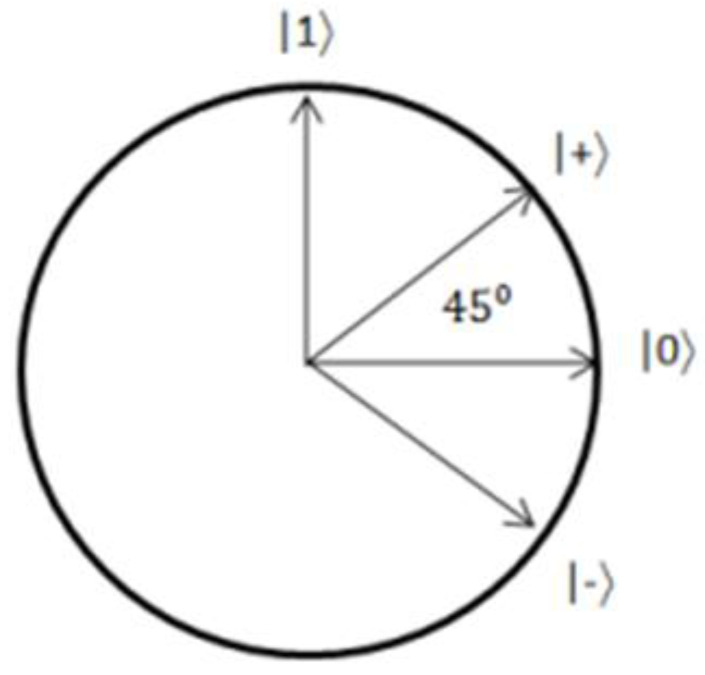
|+⟩ and |−⟩ quantum state.

The angle that was discussed is shown. It has been modified for use in the Hilbert space. The orientation of the qubits is shown in Figure 11 for an angle of 45 degrees. It continually adapts in this state, making eavesdropping incredibly difficult, even from some other quantum computer. The whole hybrid system is shown in Figure 2. The novel component of this system is its adaptability, which is accomplished via the use of quantum mechanics laws and the hybrid nature of the system. The conventional management style here rewards the whole system, creating a sense of simplicity in a complicated situation.

## 5. Comparative Discussion

Quantum cryptography, based on quantum physics and classical encryption, is a unique concept in cryptography. Quantum cryptography offers security for a variety of applications. Its primary benefits are absolute security and sniffer detection compared to conventional cryptography. These qualities can potentially resolve a significant cyberspace security issue for the future Internet.

Personal computers have been developed to a point where those unfamiliar with computer science theory might conclude there is nothing computers cannot do. While this is an understandable conclusion, it has been proven that there is a limit to the types of computation that can be performed by our classical computers, which we today consider general-purpose computers. However, in the last few decades, quantum mechanics and quantum computing have advanced, and primitive operations are now possible in the quantum sphere. Currently, encryption protocols ensure the integrity of data and identities. One such protocol is the widely adopted Diffie–Hellman protocol, an asymmetric encryption protocol that relies on the historical difficulty of factoring in large prime numbers for security.

The protocol uses public and private key pairs for each participating party. Data encrypted using one of the keys (usually a public key) can only be decrypted using the private key. A person’s public and private keys are mathematically related, but it requires factoring large prime numbers to derive the private key from the public key, which is computationally infeasible with a classical computer. Quantum computers, however, can do this in only polynomial time complexity. This development, combined with the growing power of quantum computers, gives rise to future security concerns for the Diffie–Hellman protocol.

The simulated test may be run in two ways: with and without eavesdropping. Each N value is simulated three times under identical circumstances, and the effective bits in the final key distribution process are determined by averaging the outcomes of these simulations. Error repair and key improvement are performed on the bit information that passes the security detection to guarantee the accurate and secure key for further information encryption. The symbols and meanings used in the simulation data analysis process are shown in Table 7.

The simulation test data of BB84 under different conditions are shown in Table 8.

According to the BB84 simulation test data table, when the eavesdropper Eve does not exist, the bit error rate QBERe fluctuates around 50% of the theoretical value of the BB84 protocol, and the floating range is less than the bit error rate threshold ε_0_ = 5%. The security detection is passed, and the simulation results are the same as the theoretically expected value of the protocol. When the eavesdropper Eve exists, the bit error rate fluctuates around 75%, which is close to the protocol’s theoretical value. Table 9 shows the comparison between classical key distribution and quantum key distribution channels.

Quantum cryptography will very certainly be used in cloud security. The communication technique for IoT mass customers is now more secure. More public support will be given to quantum computing applications. Because a quantum channel is nearly impossible to exploit, therefore the problem of modern hacking will be solved.

## 6. Conclusions and Future Directions

Quantum cryptosystems have generated considerable interest, and additional research on their Internet of Things application is required. This article discusses the process of developing quantum-resistant solutions for the next generation of Internet of Things developers. Quantum cryptography is a revolutionary notion in the world of cryptography. Compared to conventional encryption, its ultimate advantage is complete security and sniffer detection. These qualities can address future Internet-based cyberspace security concerns. Quantum computers may also be capable of rapidly breaking asymmetric encryption schemes based on integer factorization or discrete logarithms.

Now, the most extensively used asymmetric algorithms are based on challenging mathematical problems, such as factoring large numbers, which may take hundreds of years on today’s most powerful computers. However, Peter Shor’s study at MIT revealed over two decades ago that identical problems could potentially be solved in days or hours using a large-scale quantum computer.

This implies that we must now implement security measures to secure data protection for decades. Today’s public critical cryptography systems for protecting the keys required to encrypt data and authenticate transactions, code, and data are susceptible to future quantum computers and must be replaced. This research provided an enhanced architecture for resolving public-key cryptography challenges using SimuloQron to implement the BB84 protocol for quantum key distribution (QKD) and one-time pad (OTP). A modified version of the BB84 protocol is introduced for this IoT situation. Commercial businesses such as banks have created more efficient quantum cryptography.

On the other hand, the Internet of Things (IoT) has put millions of sensitive communications and personal devices and significant quantum computing research in danger. Quantum cryptography technologies are in high demand right now. A conceptual system was provided in this study. Further research and inquiry might lead to a more effective technique or advancement in this area.

Future fault-tolerant quantum computers may represent significant hazards, such as the capacity to break encryption protocol techniques and gain access to confidential information. To reduce these risks, IBM has defined a strategic agenda to ensure the long-term security of its platforms and service offerings. The study and development of basic quantum-safe cryptographic algorithms are part of the plan for the near future. According to IBM, “IBM now delivers the most comprehensive quantum-safe solution to data security available today to help companies safeguard present data while also preparing for future threats”.

## Figures and Tables

**Table 1:** The probability of measuring a qubit from one basis state into another.

	Measured Value				
Qubit state		|0⟩	|1⟩	|+⟩	|−⟩
	|0⟩	100%	0%	50%	50%
	|1⟩	0%	100%	50%	50%
	|+⟩	50%	50%	100%	0%
	|−⟩	50%	50%	0%	100%

**Table 2:** The effect of quantum computing on traditional cryptography techniques.

Cryptographic Algorithm	Type	Purpose	The Effects of Large-Scale Quantum Computers
Advanced Encryption Standard	Symmetric Key	Encryption	Larger key sizes are required
Secure Hash Algorithm (SHA-2 SHA-3)	Symmetric key	Hash functions	Enlarged output is required
Ron Rivest, Adi Shamir, and Len Adleman (RSA)	Public Key	A signature, key establishment	Not safe anymore
ECDSA, ECDH	Public Key	A signature, key exchange	Not safe anymore
Digital Signature Algorithm (DSA)	Public Key	A signature, key exchange	Not safe anymore

**Table 3:** Impact of quantum computing on standard cryptographic algorithms.

Symmetric Cryptography			
Name of Cryptographic Algorithm	Function	Pre-Quantum Security Level	Post-Quantum Security Level
Advanced Encryption Standard (AES-128)	Block cipher	128	64
Advanced Encryption Standard (AES-256)	Block cipher	128	64
Salsa20	Stream cipher	256	12
Galois Message Authentication Code (GMAC)	MAC	128	128
Poly1205	MAC	128	128
Secure Hash Algorithm SHA-256	Hash function	256	128
Secure Hash Algorithm 3 (SHA-3)	Hash Function	256	128
Public-Key Cryptography			
Ron Rivest, Adi Shamir, and Len Adleman (RSA 3072)	Encryption	128	Broken
Ron Rivest, Adi Shamir, and Len Adleman (RSA 3072)	Signature	128	Broken
Diffie–Hellman (DH 3072)	Key exchange	128	Broken
Digital Signature Algorithm (DSA 3072)	Signature	128	Broken
Elliptic-curve Diffie–Hellman 256-bit	Key exchange	128	Broken
Elliptic-curve Diffie–Hellman 256-bit	Signature	128	Broken

**Table 4:** The four possible states of a qubit in a BB84-encoded string.

$a_{i}$	$b_{i}$	|ψ⟩
0	0	|0⟩
1	0	|1⟩
0	1	|+⟩
1	1	|-⟩

**Table 5:** An exhaustive list of encoding and measurement of a single qubit between Alice and Bob.

Basis Alice	Basis Eve	Basis Bob	Percent Correct	Bit Kept in Key
0	0	0	100%	Kept
0	0	1	50%	Discarded
0	1	0	50%	Kept
0	1	1	50%	Discarded
1	0	0	50%	Discarded
1	0	1	50%	Kept
1	1	0	50%	Discarded
1	1	1	100%	Kept

**Table 6:** Simulation of the BB84 protocol with 10 qubits.

Qubit Number	0	1	2	3	4	5	6	7	8	9
Alice Bit Value	1	0	1	1	1	1	1	1	1	0
Alice Basis	h	H	S	h	h	h	h	h	H	S
Bob Basis	h	H	H	h	h	s	s	h	S	S
Accepted Bit Value	√	√	X	√	√	X	X	√	X	√
Alice Shifted Key	1	0		1	1			1		0
Bob Shifted Key	1	0		1	1			1		0
Tested Bits	√	√		√						

**Table 7:** Symbol representation and meaning.

Parameter Meaning	Initial Key Length	Public Survey Base	Valid Key	Security Key	Bit Error Rate
Representation method	N	Q_b_	Q_e_	Q_f_	QBER_e_

**Table 8:** BB84 simulation test data.

BB84 Simulation							
		No Eavesdroppers				Eavesdropper	
N	Q_b_	Q_e_	Q_f_	QBER_e_ (%)	Q_b_	Q_e_	QBER_e_ (%)
64	31	31	29	51.7	31	17	73.4
128	65	65	60	49.1	63	30	76.6
256	129	129	117	49.6	118	65	74.6
512	252	252	234	50.8	249	135	73.6
1024	557	557	509	51.0	507	265	74.1
2048	1033	1033	936	49.6	990	568	72.3
4096	2029	2029	1831	50.5	2054	1044	74.5

**Table 9:** Comparison of proposed architecture using quantum channel and normal key distribution using classical channel.

	Quantum Key Distribution Using Quantum Channel			Normal Key Distribution Using Classical Channel	
	With Eavesdropping	Without Eavesdropping		With Eavesdropping	Without Eavesdropping
The initial number of qubits	500	500	The initial number of bits	500	500
Final key length	106	128	Final key length	98	120
Estimated error	0.0769	0.0	Estimated error	1.25	0.5
Eavesdropping rate	1	0	Eavesdropping rate	1	0
Alice/Bob basis selection bias	0.5	0.5	Alice/Bob basis selection bias	0	0
Eve basis selection bias	0.5	0.5	Eve basis selection bias	0	0
Information leakage	83	48	Information leakage	85	53
Overall key costs for authentication	256	256	Overall key costs for authentication	324	324
Bit error probability	0.0144	0.0	Bit error probability	0.5	0.2
Security parameter	20	20	Security parameter	18	18

## Data Availability

Data can be provided on request to corresponding author.

## Abbreviations

TLS	Transport Layer Security
HTTPS	Hypertext Transfer Protocol Secure IoT: Internet of Things
OTP	One-Time Pad
QKD	Quantum Key Distribution
BB84	Bennett and Brassard 1984
SSL	Secure Sockets Layer
KEP	Key Exchange Protocol
NP-Hard	Non-deterministic Polynomial-time Hardness
IP	Internet Protocol
RSA	Rivest–Shamir–Adleman
ECC	Elliptic Curve Cryptography
DH	Diffie–Hellman
NTC	Negative Temperature Coefficient
DSA	Digital Signature Algorithm
OS	Operating System
DLP	Data Loss Prevention Software
